# Sensitization of estrogen receptor-positive breast cancer cell lines to 4-hydroxytamoxifen by isothiocyanates present in cruciferous plants

**DOI:** 10.1007/s00394-015-0930-1

**Published:** 2015-05-27

**Authors:** Anna Pawlik, Monika Słomińska-Wojewódzka, Anna Herman-Antosiewicz

**Affiliations:** Department of Molecular Biology, University of Gdańsk, Wita Stwosza 59, 80-308 Gdańsk, Poland

**Keywords:** Breast cancer, Sulforaphane, Erucin, 4-Hydroxytamoxifen, Estrogen receptor

## Abstract

**Purpose:**

Tamoxifen has been used for the treatment of estrogen receptor (ER)-positive breast cancers and in women who are at an increased risk of breast cancer. Acquired resistance to this drug and its toxicity still pose a clinically significant problem, especially in the prevention setting. Isothiocyanates present in cruciferous plants, such as sulforaphane or erucin, have been shown to reduce growth of breast cancer cells in vivo and in vitro. In this study, we explored their ability to sensitize cancer cells to 4-hydroxytamoxifen.

**Methods:**

We used three ER-positive breast cancer cell lines, T47D, MCF-7 and BT-474, as well as the drug-resistant T47D and MCF-7 derivatives. We examined the effect of 4-hydroxytamoxifen, isothiocyanates and their combinations on cell viability by MTT and clonogenic assays. Impact of treatments on the levels of proteins engaged in apoptosis and autophagy was determined by Western blotting.

**Results:**

Isothiocyanates act in a synergistic way with 4-hydroxytamoxifen, and co-treatment reduces breast cancer cell viability and clonogenic potential more effectively than treatment with any single agent. This is connected with a drop in the Bcl-2/Bax ratio and the level of survivin as well as increased PARP cleavage, and elevation in ADRP, the mitochondrial stress marker. Moreover, isothiocyanates sensitize 4-hydroxytamoxifen-resistant T47D and MCF-7 cells to the drug.

**Conclusion:**

Isothiocyanates enhance response to 4-hydroxytamoxifen, which allows for reduction of the effective drug concentration. Combinatorial strategy may hold promise in development of therapies and chemoprevention strategies against ER-positive breast tumors, even those with acquired resistance to the drug.

## Introduction

Breast cancer is the leading cause of cancer-related deaths in women [27]. Estrogen receptor (ER)-positive breast tumors comprise approximately 75 % of the breast cancer cases [6]. Tamoxifen is a drug conventionally used in prevention and treatment of advanced estrogen receptor-positive breast cancer in pre- and postmenopausal women [13, 58]. Tamoxifen and its active metabolite, 4-hydroxytamoxifen, act as an estrogen antagonist or agonist, depending on tissue and organ type. The ER, a regulator of expression of genes involved in the ER-positive tumor progression, is able to stimulate cancer growth in two different ways: a classical way by binding to its responsive elements in a given gene’s promoter, and nongenomic through activation of growth factor receptors and cellular pro-survival kinases [8, 43]. 4-Hydroxytamoxifen demonstrates estrogen antagonist activity in breast cells. All ER antagonists display the same crucial mechanism of action: They bind ER and block its activity [17, 28]. However, many breast cancer cells show a primary or secondary endocrine resistance. Statistics indicates that in 30–50 % of women with ER-positive breast cancer, de novo or intrinsic resistance to tamoxifen occurs and in many patients tumor recurrence is observed after drug therapy. Different molecular mechanisms may lead to a development of cellular resistance to the hormone therapy and protect cancer cells from death induced by drugs. Among them, the loss of ER or alteration in its structure and function, overactivation of serine/threonine protein kinase B (Akt), alteration in the ER signal transduction, and crosstalk between the ER and growth factor receptors have been reported [50, 51]. Moreover, long-term administration of the selective ER modulator may lead to serious side effects, such as menopausal symptoms, venous thromboembolic events, endometrial hyperplasia, polyps and cancer or ovarian cysts [41, 42].

Isothiocyanates (ITC) are naturally occurring phytochemicals present in cruciferous plants. Sulforaphane [1-isothiocyanato-4-(methylsulfinyl)-butane), SFN] and its reduced analog, erucin [1-isothiocyanato-4-(methylthio)-butane, ERN], exhibit chemopreventive and antitumor activities against different types of cancers. The molecular mechanisms of SFN action include inhibition of phase I carcinogen-activating enzymes, induction of phase II carcinogen detoxification enzymes, and induction of the cell cycle arrest and apoptosis. Preventive activity of SFN has been reported in numerous in vivo models. Administration of SFN by oral gavage inhibited development of mammary tumors in female Sprague–Dawley rats treated with the 9,10-dimethyl-1,2-benzanthracene (DMBA) carcinogen [65] or prostate carcinogenesis and pulmonary metastasis in TRAMP mice [57]. Feeding A/J mice with SFN and its *N*-acetylcysteine conjugate resulted in an inhibition of malignant progression of lung adenomas induced by the tobacco carcinogens [15]. Sulforaphane protected male Syrian hamsters treated with the pancreatic carcinogen, *N*-nitroso-bis(2-oxopropyl)amine, against the development of a pancreatic tumor [35]. These observations were supported by in vitro studies, showing that SFN induces apoptosis, for instance, in PC-3 prostate cancer cells [56], MDA-MB-231, MDA-MB-468, MCF-7 and T47D human breast cancer cell lines [49], UM-UC-3 bladder tumor cells [59], A549 nonsmall lung cancer cells [40] and HT29 colon cancer cell lines [19]. SFN has been also shown to inhibit cell cycle progression of different cancer cells, including PC-3 prostate cancer cells [55], HT29 human colon cancer cells [48, 53], PaCa-2 and PANC-1 pancreatic cancer cells [48] or MCF-7 breast cancer cells [24]. In addition, it has been shown that SFN induces autophagy [22, 31]. In the case of cells with the defective apoptosis, autophagy may lead to the type II programmed cell death. In some conditions, however, it may suppress or delay cell death, such as in the case of prostate or breast cancer cells treated with SFN [22, 31].

Erucin is abundant in salad rocket and can be generated by interconversion of SFN. It has been reported that erucin also modulates phase I enzymes [37] and phase II enzymes [26], induces pro-apoptotic signals and influences cell cycle progression, for instance, in human leukemia cells [25], HepG2 human hepatocellular carcinoma cells [36], human lung carcinoma A549 cells [39], bladder J82, UM-UC-3 cancer cells [1] and more recently—in MCF-7 breast cancer cells [5].

On the basis of the data described above as well as recently reported results showing that SFN inhibits pro-survival Akt-mTOR-S6K pathway in phenotypically different breast cancer cells [45], we hypothesize that application of ITC may enhance anti-proliferative activity of 4-hydroxytamoxifen. In the present study, we used three ER-positive breast cancer cell lines, T47D, MCF-7 and BT-474, as well as drug-resistant derivatives of T47D and MCF-7 cells, to compare their sensitivity to structurally related ITC (sulforaphane and erucin) as well as 4-hydroxytamoxifen, and test whether these naturally occurring phytochemicals sensitize ER-positive breast cancer cells to the drug.

## Materials and methods

### Reagents

R,S-sulforaphane (purity ≥ 98 %) and erucin (purity ≥ 98 %) were obtained from LKT Laboratories (St. Paul, MN). They were prepared in DMSO and stored at a stock concentration of 10 mM at −20 °C. RPMI-1640 medium, fetal bovine serum was purchased from Life Technologies (Grand Island, NY). (Z)-4-hydroxytamoxifen (purity ≥ 98 %), penicillin–streptomycin solution, DMSO, sulforhodamine B, 3-(4,5-dimethylthiazol-2-yl)-2,5-diphenyltetrazolium bromide (MTT), anti-rabbit and anti-β-actin antibodies conjugated with HPR were from Sigma-Aldrich (St. Louis, MO). (Z)-4-hydroxytamoxifen (purity ≥ 98 %) was dissolved in ethanol at a concentration of 10 mM and stored at 4 °C. Antibodies against Bcl-2, Bax, survivin and ADRP were from Santa Cruz Biotechnology (Santa Cruz, CA), antibody against PARP was from Cell Signaling Technology (Danvers, MA) and anti-LC3 antibody was purchased from Medical and Biological Laboratories Co., Ltd. (Woburn, MA).

### Cell culture and treatment

ER-positive breast cancer cell lines, MCF-7, T47D and BT-474, were cultured in RPMI-1640 supplemented with 10 % fetal bovine serum and 1 % penicillin/streptomycin solution. Tamoxifen-resistant derivatives of MCF-7 and T47D cells were obtained by continuous exposure of parental cell lines to increasing concentrations of 4-hydroxytamoxifen for over 15 months. Cells were cultured in RPMI medium without phenol red, with 10 % charcoal stripped-fetal bovine serum. For the first 3 months, parental cells were continuously treated with 100 nM 4-hydroxytamoxifen. After this time, the concentration of the drug was increased to 500 nM. The medium with the appropriate concentration of 4-hydroxytamoxifen was changed every 4 days. ER-negative breast cancer cell line, MDA-MB-231, was maintained in MEM supplemented with 10 % FBS, 1 mM sodium pyruvate, nonessential amino acids and antibiotics. Each cell line was incubated at 37 °C in 5 % CO_2_. Twenty four hours after plating, cells were treated with the desired concentration of isothiocyanate with or without (Z)-4-hydroxytamoxifen or an equivalent volume of ethanol and/or DMSO (vehicle control) for 96 h with medium replacement after 48 h.

### Cell viability assay

Cells were seeded at density 2 × 10^3^ cells per well in 96-well plate and allowed to grow for 24 h. After that time cells were treated with increasing concentrations of ITC (2.5–50 μM), increasing concentrations of 4-hydroxytamoxifen (0.05–10 μM) or combinations of both. Control samples were treated with DMSO, ethanol or DMSO and ethanol at appropriate concentrations. After 48 h, medium was replaced with the fresh medium containing the same supplements. After 96 h of treatment, 25 μl of MTT (3-(4,5-dimethylthiazol-2-yl)-2,5-diphenyltetrazolium bromide) stock solution (4 mg/ml in PBS) was added to each well for 4 h. Next, the formazan crystals were dissolved in 100 % DMSO, and absorbance was measured at 570 nm with a reference filter of 660 nm in Victor3 microplate reader (PerkinElmer Life and Analytical Sciences, Boston, MA). To inhibit autophagy induction, cells were pretreated with 50 nM wortmannin and then treated with ITC and 4-hydroxytamoxifen as described above. After 96 h, 100 μl of 10 % (w/v) aqueous solution of ice-cold trichloroacetic acid was added for 1 h. Plates were washed with water, allowed to air-dry and stained with 100 μl of 0.4 % sulforhodamine B solution in 1 % acetic acid for 15 min. Cells were washed 5 times with 1 % acetic acid and dried. After addition of 10 mM Tris base (pH 10.5, 150 μl/well), the absorbance was measured at 570 nm using Victor3 microplate reader. Data were obtained from at least three independent experiments, and each treatment condition assayed in triplicate.

### Clonogenic assay

Cells were plated at density 5 × 10^4^ per 100 mm plate. After 24 h, cells were treated with 0.5 μM 4-hydroxytamoxifen, ITC or combinations of these chemicals. Control samples were treated with ethanol and/or DMSO at appropriate concentration for 8 days, and every 2 days the medium was changed to the fresh one with the same supplements. After 8 days of treatment, cells were trypsinized and plated at 800 per 100 mm plate in duplicate. Colonies were stained with crystal violet (0.5 % w/v) and counted 2 weeks (in case of T47D and MCF-7 cell lines) or 4 weeks (in case of BT-474 cells) after plating. The experiment was repeated twice.

### Western blot analysis

MCF-7 and T47D cells were plated at density 2.5 × 10^5^ and BT-474 at density 5 × 10^5^ per 100 mm plate. Cells were treated as described in the “Cell culture and treatment” section. Control samples were treated with ethanol and/or DMSO at appropriate concentrations. After 48 h, medium was changed to fresh one and the compounds were added again. After 96 h, cells were lysed in a solution containing 50 mM Tris (pH 7.5), 1 % Triton X-100, 150 mM NaCl, 0.5 mM EDTA, protease and phosphatase inhibitor cocktails (Roche Diagnostics, Germany) and centrifuged at 13,000 rpm at 4 °C for 30 min. Immunoblots were performed as previously described [45].

### Statistical analysis and analysis of synergy

Data were analyzed using GraphPad Prism software. One-way ANOVA followed by Bonferroni’s multiple comparison test was used to determine statistical significance of differences in the measured variables. Differences were considered significant at *p* < 0.05.

Data from MTT viability assay were analyzed using the method of Chou and Talalay [11] and CompuSyn software to determine the dose that gives the median effect, linear correlation coefficient in case of treatment with a single compound, and combination index (CI) for samples treated with two compounds. The CI is a quantitative measure of the degree of interaction between drugs. CI < 1, CI = 1 and CI > 1 denote synergism, additivity and antagonism, respectively. Variable ratios of ITC and 4-hydroxytamoxifen were used.

## Results

### Effect of ITC, 4-hydroxytamoxifen or their combinations on survival of ER-positive breast cancer cell lines

**Fig. 1:**
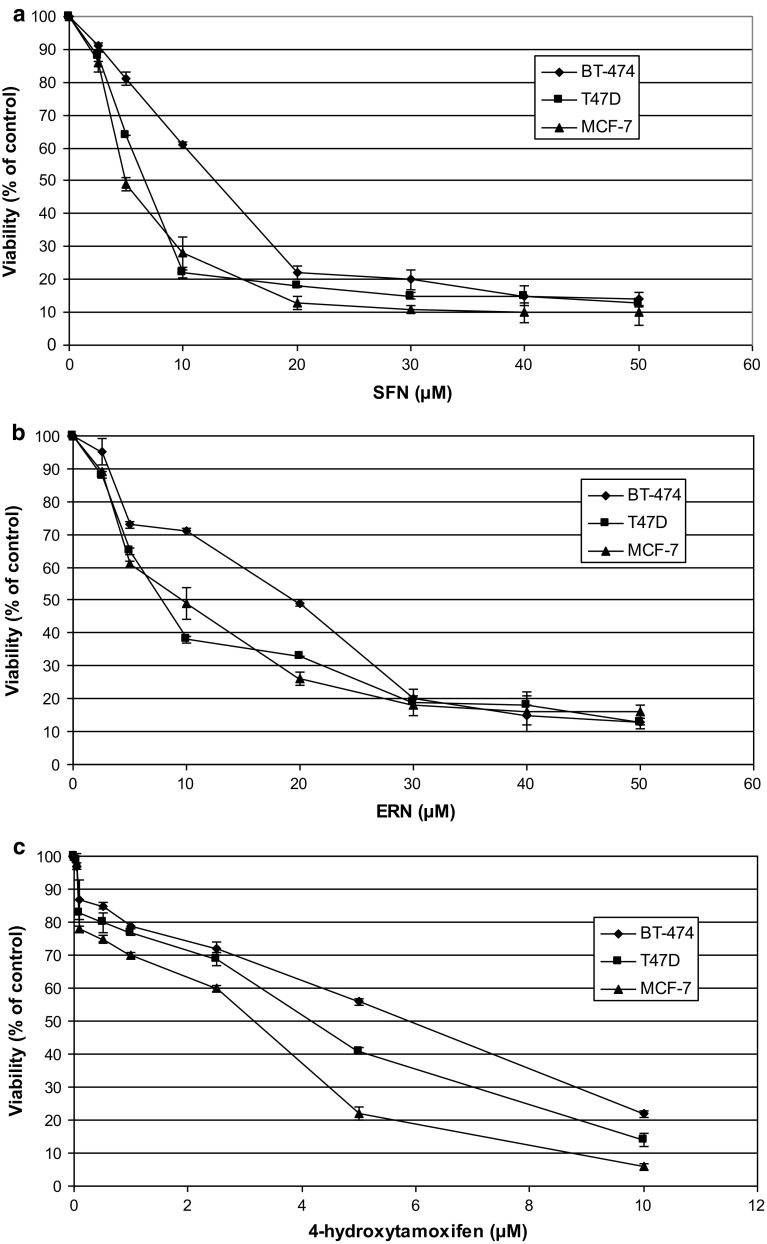
Dose-dependent effect of SFN (**a**), ERN (**b**) or 4-hydroxytamoxifen (**c**) on survival of T47D, MCF-7 and BT-474 cell lines after 96 h of treatment. Control samples were treated with appropriate concentrations of DMSO. Viability was assayed by MTT method as described in “Materials and methods”. Results shown are mean ± SE of three independent experiments performed in triplicate

We investigated the effect of sulforaphane, erucin and 4-hydroxytamoxifen alone or in combinations on viability of three ER-positive breast cancer cell lines: MCF-7, T47D and BT-474. Both ITC inhibited cell growth in a dose-dependent manner (Fig. 1). T47D and MCF-7 cells were similarly sensitive to sulforaphane (IC_50_ were 6.6 and 5 μM, respectively) and to erucin (IC_50_ = 7.6 and 9.7 μM, respectively). Interestingly, marked differences in sensitivity of BT-474 cell line to sulforaphane and erucin were observed with IC_50_ values of 15 or 19.7 μM, respectively (Fig. 1a, b). Sensitivity of T47D, MCF-7 and BT-474 cell lines to 4-hydroxytamoxifen was dependent of its dose, and IC_50_ values after 96 h of treatment were 4.2, 3.2 and 5.7 μM, respectively (Fig. 1c).

**Fig. 2:**
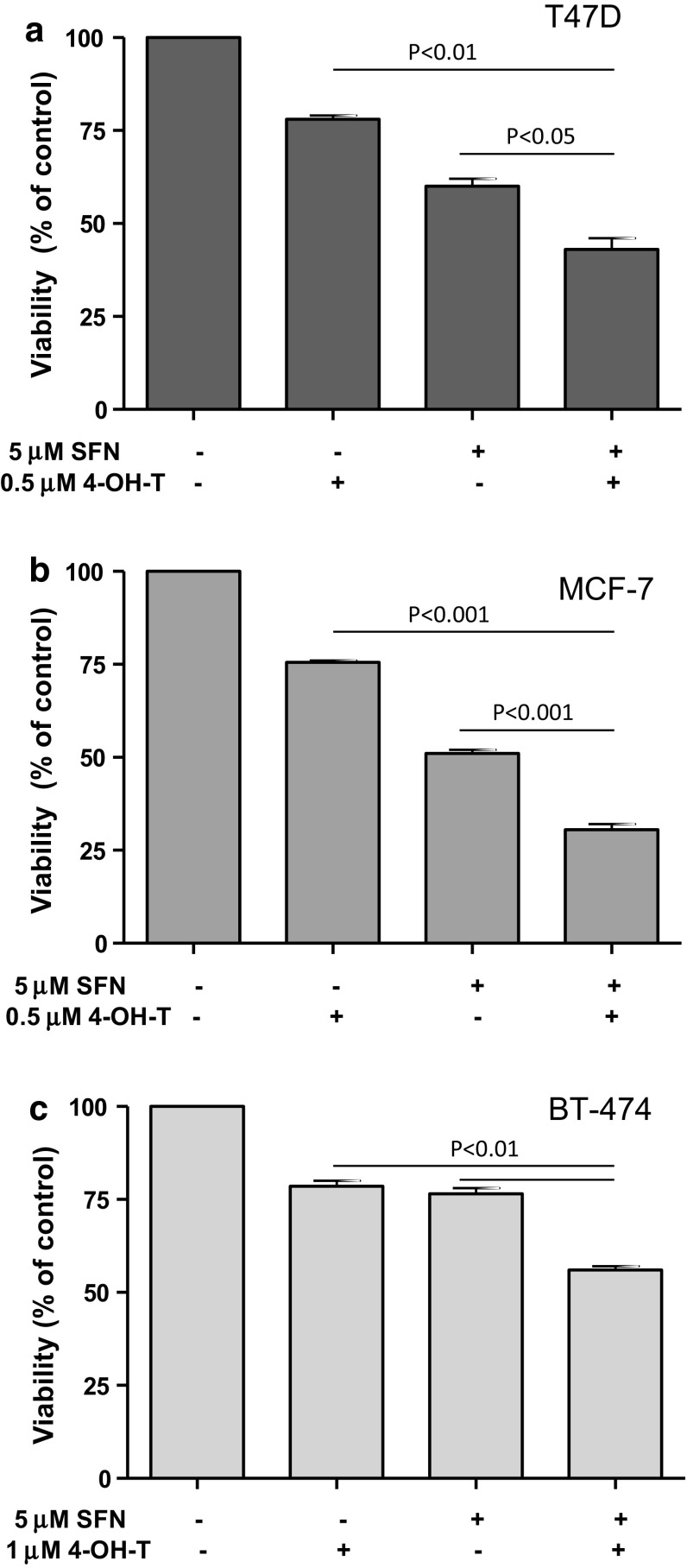
Effect of 96-h treatment with sulforaphane (SFN, 5 μM), 4-hydroxytamoxifen (4-OH-T: 0.5 μM in **a** and **b** or 1 μM in **c**) or both compounds on viability of T47D (**a**), MCF-7 (**b**) and BT-474 cells (**c**). Viability was assayed by MTT method as described in “Materials and methods”. Results shown are mean ± SE of three independent experiments performed in triplicate. *p* values were calculated by one-way ANOVA followed by Bonferroni’s multiple comparison test

**Fig. 3:**
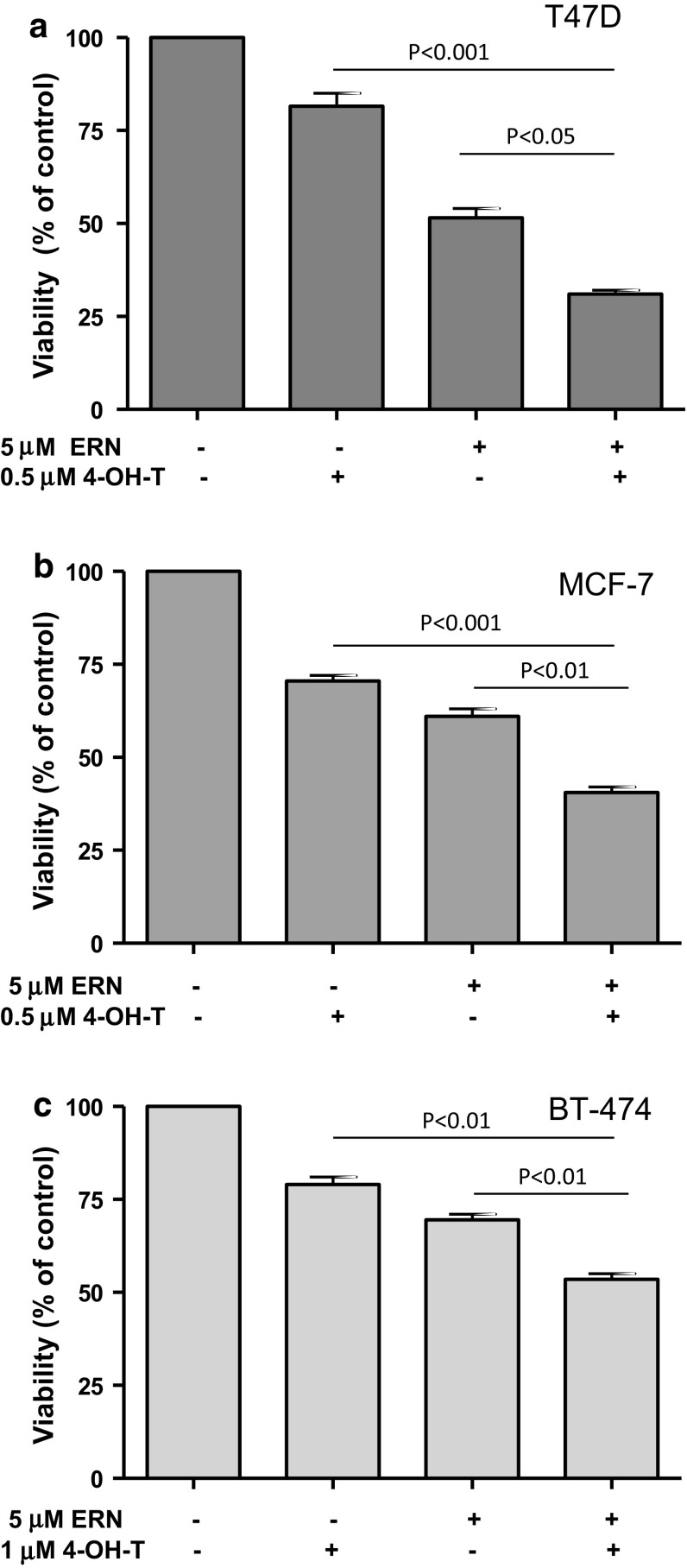
Effect of 96-h treatment with erucin (ERN, 5 μM), 4-hydroxytamoxifen (4-OH-T: 0.5 μM in **a** and **b** or 1 μM in **c**) or both compounds on viability of T47D (**a**), MCF-7 (**b**) and BT-474 cells (**c**). Results shown are mean ± SE of three independent experiments performed in triplicate. *p* values were calculated by one-way ANOVA followed by Bonferroni’s multiple comparison test

Next, we assessed viability of three breast cancer cell lines after co-treatment with ITC at concentrations equal or lower than their respective IC_50_ value (5 μM) and 4-hydroxytamoxifen at a concentration close to its IC_20_ (0.5 or 1 μM). The results obtained showed that combination of ITC and 4-hydroxytamoxifen inhibited cell viability more efficiently than either compound used alone: It was about 20 % lower upon combined treatment than viability of cells treated with ITC and about 30–50 % lower than viability of cells treated with 4-hydroxytamoxifen only (Figs. 2, 3). To elucidate whether the greater effect of combined treatment than mono-treatment is due to synergistic action of the used compounds, we calculated a combination index (CI) for the nonconstant ratio combinations using the method of Chou and Talaly and data from the MTT viability assays (linear correlation coefficient for each drug in the growth inhibition plot was >0.9). As shown in Table 1, CI values for tested drug combinations were below 1, which indicates synergism.

**Table 1:** Combination indexes of sulforaphane (SFN) or erucin (ERN) and 4-hydroxytamoxifen (4-OH-T) in breast cancer cells. CI < 1 indicates synergism

Cell line	Combination	CI
T47D	SFN (5 μM) + 4-OH-T (0.5 μM)	0.71
	ERN (5 μM) + 4-OH-T (0.5 μM)	0.36
MCF-7	SFN (5 μM) + 4-OH-T (0.5 μM)	0.63
	ERN (5 μM) + 4-OH-T (0.5 μM)	0.66
BT-474	SFN (5 μM) + 4-OH-T (1 μM)	0.81
	ERN (5 μM) + 4-OH-T (1 μM)	0.61

### Effect of isothiocyanate and 4-hydroxytamoxifen co-treatment on apoptosis induction in the analyzed breast cancer cell lines

**Fig. 4:**
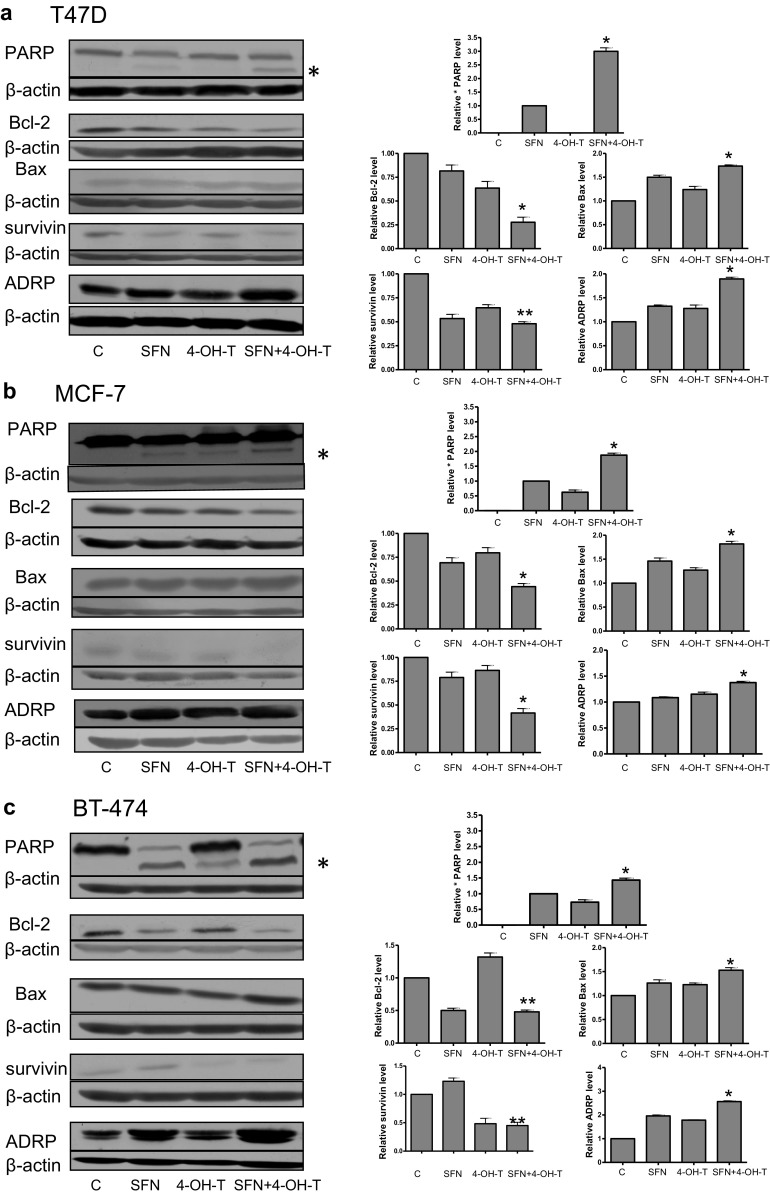
Effect of co-treatment of breast cancer cell lines with 4-hydroxytamoxifen and sulforaphane on PARP cleavage and levels of Bcl-2, Bax, survivin and ADRP. T47D (**a**) and MCF-7 (**b**) cells were treated with 5 μM sulforaphane (SFN), and/or 0.5 μM 4-hydroxytamoxifen (4-OH-T). BT-474 (**c**) cells were treated with 5 μM sulforaphane (SFN) and/or 1 μM 4-hydroxytamoxifen (4-OH-T). *Blots* were stripped and reprobed with anti-β-actin antibody to ensure equal protein loading. Results are plotted as mean ± SE from three independent experiments, *significantly different compared with single agent-treated samples or **significantly different compared with one of the single agent-treated samples by one-way ANOVA followed by Bonferroni’s multiple comparison test. Data for PARP refer to the faster migrating band marked as * and are given relative to samples treated with SFN alone. *Blots* shown are representative of at least three independent experiments

**Fig. 5:**
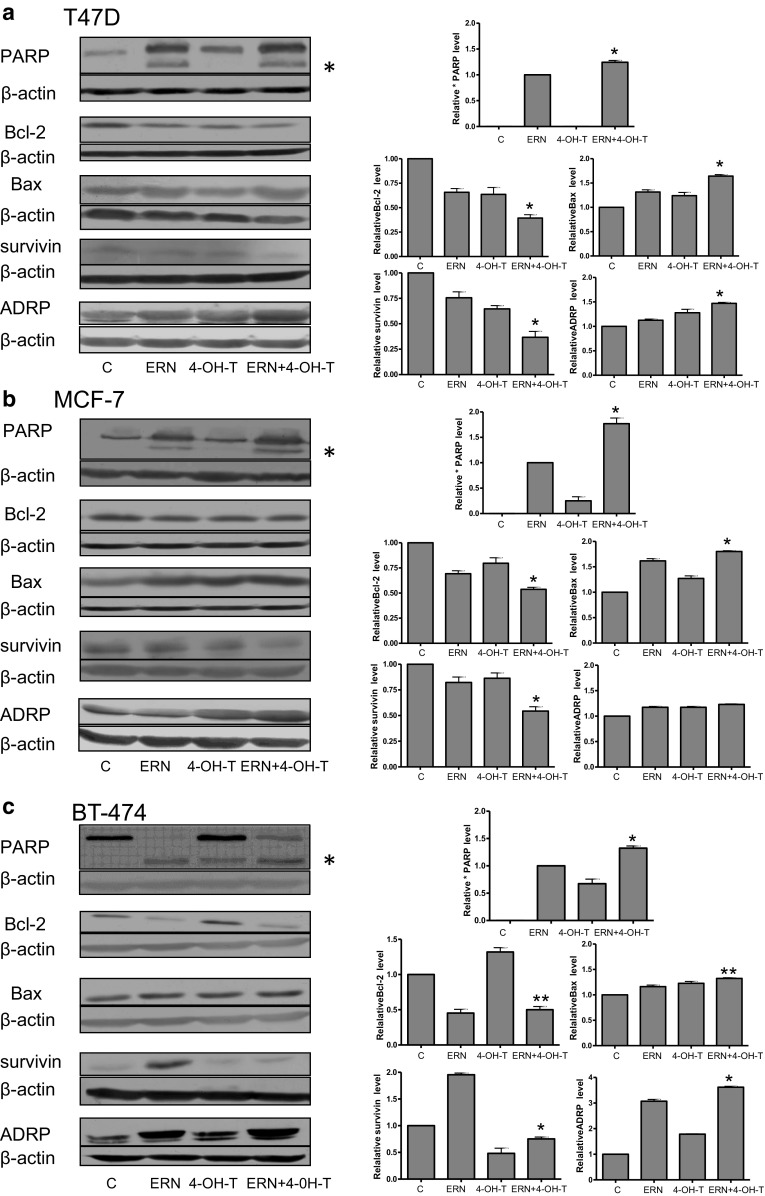
Effect of co-treatment of breast cancer cell lines with 4-hydroxytamoxifen and erucin on PARP cleavage, levels of Bcl-2, Bax, survivin and ADRP. T47D (**a**) and MCF-7 (**b**) cells were treated with 5 μM erucin (ERN) and/or 0.5 μM 4-hydroxytamoxifen (4-OH-T). BT-474 (**c**) cells were treated with 5 μM erucin (ERN) and/or 1 μM 4-hydroxytamoxifen (4-OH-T). *Blots* were stripped and reprobed with anti-β-actin antibody to ensure equal protein loading. Results are plotted as mean ± SE from 3 independent experiments, *significantly different compared with single agent-treated samples or **significantly different compared with one of the single agent-treated samples by one-way ANOVA followed by Bonferroni’s multiple comparison test. Data for PARP refer to the faster migrating band marked as * and are given relative to samples treated with ERN alone. *Blots* shown are representative of at least three independent experiments

To elucidate whether decreased viability of breast cancer cells treated with the combination of ITC and 4-hydroxytamoxifen results from an apoptosis induction, we compared caspase-dependent cleavage of PARP in control cells as well as in cells treated with ITC and/or the drug. The immunoblotting analysis showed that 4-hydroxytamoxifen at 0.5 μM in T47D cells had no effect on PARP status while at 0.5 μM in MCF-7 cells and at 1 μM concentration in BT-474 cell line had a minimal effect on apoptosis induction as assessed by PARP cleavage (Figs. 4, 5). Sulforaphane and erucin at 5 μM concentrations induced PARP cleavage with intensity dependent on the cell line. However, combined treatment with SFN or ERN and 4-hydroxytamoxifen further elevated PARP cleavage (Figs. 4, 5).

It has been previously reported that ITC induce apoptosis mainly through the mitochondrial pathway; thus, we determined the level of anti-apoptotic Bcl-2 and pro-apoptotic Bax upon treatment with ITC and/or the drug. As shown in Figs. 4, 5, combinations of SFN or ERN with 4-hydroxytamoxifen decreased the Bcl-2 level most efficiently (to 30–50 % of the level seen in control cells), while the Bax level was elevated (about 50 % above the level seen in controls). Thus, reduction of Bcl-2/Bax ratio in cells treated with combinations of compounds might lead to mitochondria-mediated induction of apoptosis. As mitochondrial dysfunction may trigger formation of lipid droplets, we determined the level of adipocyte differentiation-related protein (ADRP) which decorates membranes of these organelles. As can be seen in Figs. 4 and 5, the ADRP level was elevated in cells treated with SFN or ERN and 4-hydroxytamoxifen when compared with cells treated with a single compound. Finally, the level of survivin, which is an inhibitor of caspase 3, 7 and 9, and is a mitosis promoter, was efficiently reduced by combined treatment as compared to controls and a single compound treatment, excluding BT-474 cells, where ERN alone increased survivin level about 100 % above control, and although combination with 4-hydroxytamoxifen lowered its amount, it was still higher than in the drug-only-treated cells (Fig. 4).

### Impact of the co-treatment of T47D, MCF-7 and BT-474 cells with 4-hydroxytamoxifen and isothiocyanates on induction of autophagy

**Fig. 6:**
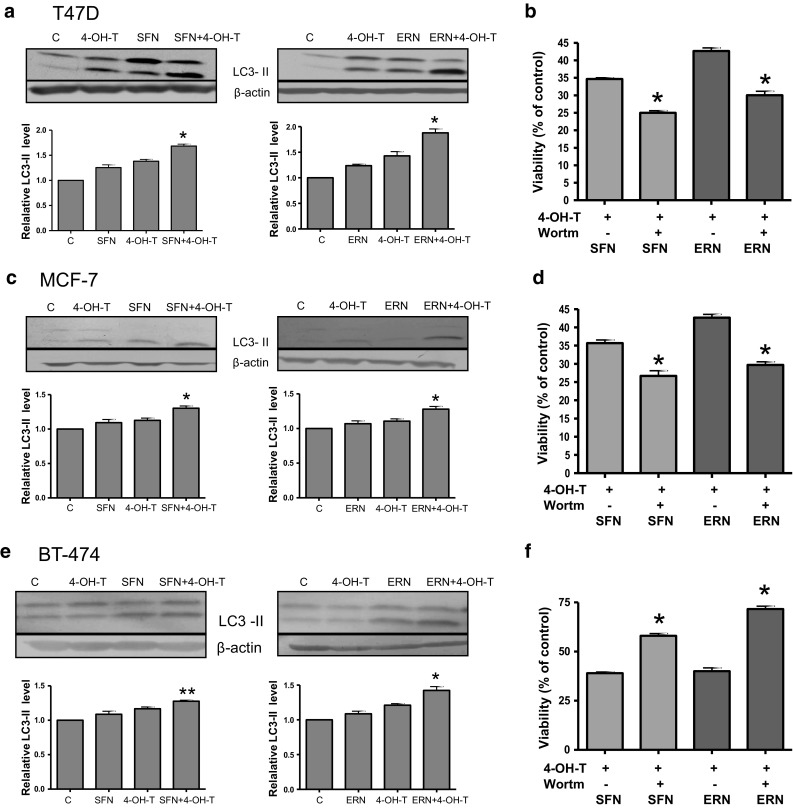
Autophagy process in T47D (**a**, **b**), MCF-7 (**c**, **d**) and BT-474 cells (**e, f**) treated with sulforaphane, erucin, 4-hydroxytamoxifen or combinations. T47D and MCF-7 cells were treated with 5 μM sulforaphane (SFN), 5 μM erucin (ER) and/or 0.5 μM 4-hydroxytamoxifen (4-OH-T), and BT-474 cells were treated with 5 μM SFN, 5 μM ERN and/or 1 μM 4-OH-T. **a**, **c**, **e** LC3 processing (autophagy marker) was determined by immunoblotting. The *blots* were stripped and reprobed with anti-β-actin antibody to ensure equal protein loading. Results are plotted as mean ± SE from 3 independent experiments, *significantly different compared with both single agent-treated samples or **significantly different compared with one of the single agent-treated samples by one-way ANOVA followed by Bonferroni’s multiple comparison test. **b**, **d**, **f** Impact of autophagy on viability of cells treated with indicated compounds was assessed by SRB method upon autophagy inhibition by 5 nM wortmannin. *p < 0.001

Numerous studies have shown that MCF-7 and T47D cells undergo autophagy under adverse conditions, such as tamoxifen treatment. We investigated whether ITC induce autophagy in these cells and whether co-treatment with 4-hydroxytamoxifen and ITC potentiates this process. We analyzed conversion of soluble LC3-I to the lipid-bound LC3-II form which is an established marker of autophagy. As can be seen in Fig. 6a, c, clear intensification of LC3 processing (increased level of LC3-II and decreased level of LC3-I) was observed in T47D and MCF-7 cells treated with combination of compounds. In BT-474 cells, combination of ITC and 4-hydroxytamoxifen slightly increased LC3-II, while the LC3-I form was still abundant as compared with control cells or cells treated with either drug alone (Fig. 6e). To reconcile if autophagy induced by the investigated compounds plays pro-survival or pro-death role, we applied wortmannin which inhibits first stages of this process and determined viability of cells. As shown in Fig. 6b, d, inhibition of autophagy induced by combination of SFN or ERN and 4-hydroxytamoxifen in T47D and MCF-7 cells reduced their viability, which suggests that in these cell lines, autophagy plays a protective role. On the contrary, BT-474 cells revealed increased survival upon treatment with combinations when autophagy was inhibited (Fig. 6f), which suggests that in this case, autophagy contributes to cell death.

### Combined treatment decreases ability of cells to proliferate indefinitely

**Fig. 7:**
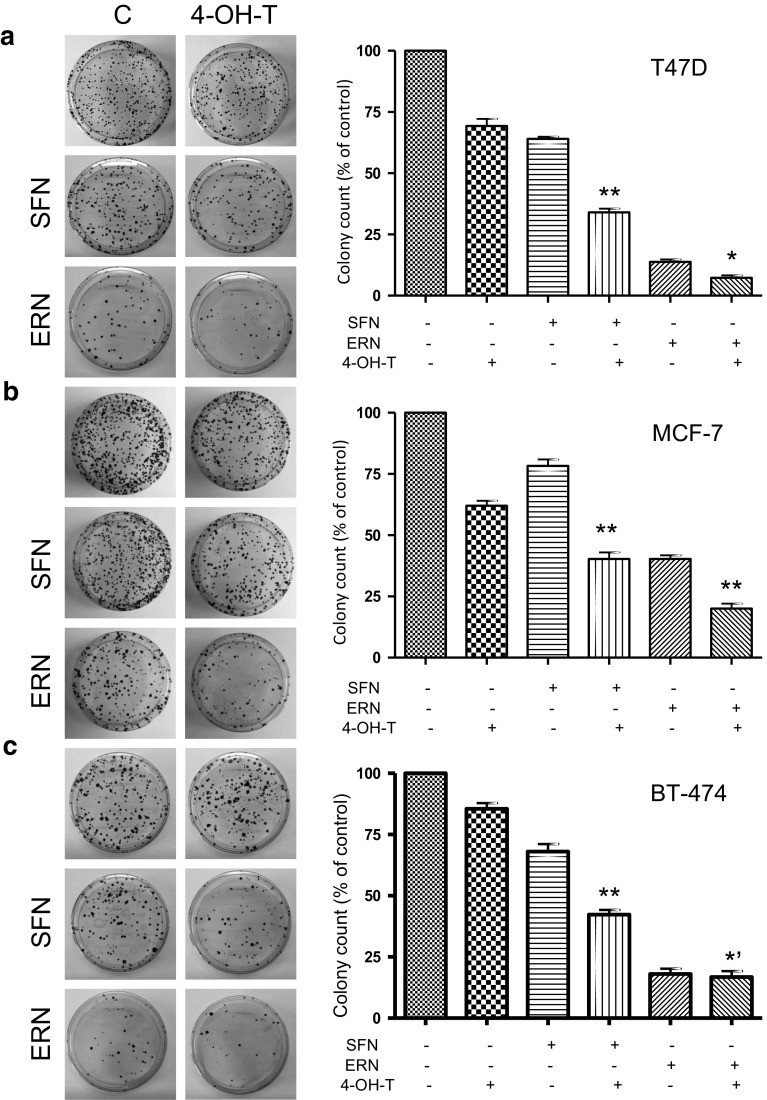
Effect of treatment with sulforaphane (SFN), erucin (ERN), 4-hydroxytamoxifen (4-OH-T) or combination of isothiocyanate and the drug on clonogenic potential of T47D (**a**), MCF-7 (**b**) and BT-474 cells (**c**). Cells were exposed to 5 μM SFN, 5 μM ERN and/or 0.5 μM (**a**, **b**) or 1 μM (**c**) 4-hydroxytamoxifen for 8 days. After that time, cells were replated at lower confluences and allowed to growth for 2 (T47D and MCF-7) or 4 weeks (BT-474) in drug-free medium. Colonies arisen from cells retaining proliferative potential were counted, and results are shown as mean ± SE of two independent experiments performed in duplicate. Statistical significance of difference was determined by one-way ANOVA followed by Bonferroni’s multiple comparison test, and **p < 0.001 as compared to ITC and 4-OH-T alone, *p < 0.001 as compared to 4-OH-T only and *p* < 0.05 as compared to erucin only, *p < 0.001 as compared to 4-OH-T only and *p* > 0.05 as compared to erucin only

As one of the main problems in anticancer therapies is recurrence of the disease, effective treatment should irreversibly eliminate cancer cells. Thus, we assessed the clonogenic potential of breast cancer cells treated with 4-hydroxytamoxifen and/or ITC for 8 days and allowed for their recovery in a drug-free medium for 2–4 weeks. Results presented in Fig. 7 clearly show that clonogenic potential of cells treated with combination of 4-hydroxytamoxifen and SFN is significantly lower than in the case of cells treated with each agent alone. Moreover, combined treatment reduces clonogenicity compared to controls by about 60 % (MCF-7 and BT-474 cells) or 70 % (T467D cells). Erucin per se is a highly cytotoxic agent (reduces clonogenic potential compared to controls to 40 % in MCF-7 cells and 14–18 % in T47D and BT-474 cells). Its combination with 4-hydroxytamoxifen potentiates cytotoxicity in MCF-7 and T47D cell lines by about twofold (Fig. 7a, b), while has no further effect on clonogenicity of BT-474 cells (Fig. 7c).

### Isothiocyanates sensitize tamoxifen-resistant ER-positive breast cancer cells to the drug

**Fig. 8:**
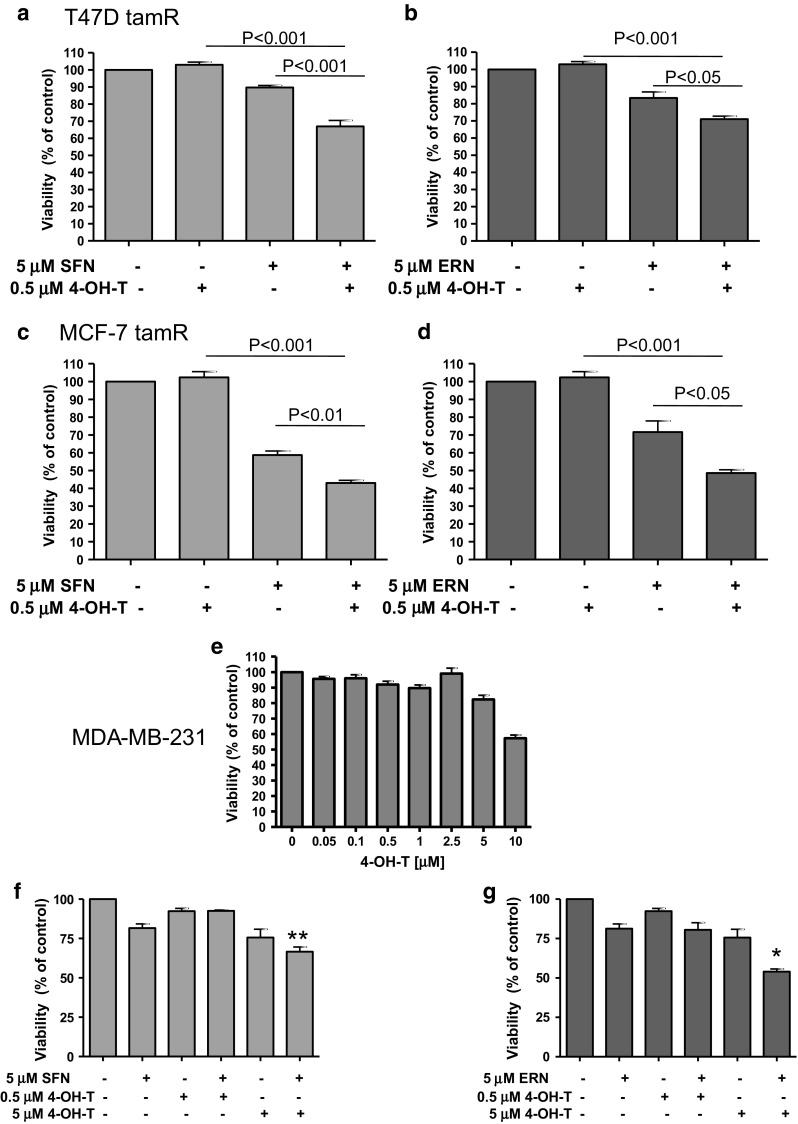
Isotiocyanates sensitize to 4-hydroxytamoxifen drug-resistant cells. Tamoxifen-resistant T47D (T47D tamR) and MCF-7 (MCF-7 tamR) derivatives were obtain as described in “Materials and methods”. Viability of T47D tamR (**a**, **b**) or MCF-7 tam R (**c**, **d**) after 96-h treatment with sulforaphane (SFN, 5 μM), erucin (ERN, 5 μM) or/and 4-hydroxytamoxifen (4-OH-T: 0.5 μM) was assessed by MTT assay. **e**–**g** Viability of ER-negative MDA-MB-231 cells was determined after treatment with increasing concentrations of 4-hydroxytamoxifen (4-OH-T) (**e**), SFN (5 μM) and/or 4-OH-T (0.5 μM or 5 μM) (**f**), ERN (5 μM) and/or 4-OH-T (0.5 μM or 5 μM) (**g**). Results shown are mean ± SE of three independent experiments performed in triplicate. *Significantly different compared with both single agent-treated samples or **significantly different compared with SFN-only-treated samples by one-way ANOVA followed by Bonferroni’s multiple comparison test

As acquired resistance to tamoxifen occurs frequently, we decided to verify whether ITC affect sensitivity of tamoxifen-resistant cells to this drug. Thus, we established T47D and MCF-7 derivatives (T47D tamR and MCF-7 tamR, respectively), which were able to grow in media containing 500 nM of 4-hydroxytamoxifen (Fig. 8a–d) and retained ER expression (data not shown). T47D tamR cell line also appeared to be less sensitive to sulforaphane or erucin than parental cell line (Fig. 8a, b). Importantly, ITC sensitized tamoxifen-resistant cells to the drug: Combination of SFN or ERN with 500 nM of 4-hydroxytamoxifen caused a statistically significant drop in viability of the cells tested as compared to the treatment with any single compound (Fig. 8a–d).

It has been shown that sulforaphane effectively lowers viability of both ER-positive and ER-negative breast cancer cells [45, 49]. To determine whether SFN or ERN sensitize cells to 4-hydroxytamoxifen in an ER-independent manner, we used ER-negative MDA-MB-231 cell line, which appeared resistant to the drug at concentrations used by us for ER-positive cells (0.5 and 1 μM) (Fig. 8e). Combination of 5 μM sulforaphane or erucin with 500 nM of 4-hydroxytamoxifen did not potentiate sensitivity of MDA-MB-231 cells to any single agent. However, we observed enhanced activity of the combination of ITC and 4-hydroxytamoxifen toward ER-negative cells when 4-hydroxytamoxifen was applied at higher, cytotoxic concentration (5 μM) (Fig. 8f, g). Combination of ERN with 5 μM 4-hydroxytamoxifen reduced viability of MDA-MB-231 more efficiently than any compound used alone (Fig. 8g).

## Discussion

Epidemiological studies indicate that frequent intake of cruciferous vegetables rich in ITC may reduce the risk for developing cancers [60]. Studies performed with laboratory animals and cancer cell lines confirmed this notion and gave insight into molecular mechanism of not only chemopreventive but also anticancer activities of these phytochemicals (for review, see [21]). It has been previously documented for different breast cancer cell lines that sulforapahane downregulates ER-α, EGFR and HER2 proteins [49] as well as PI3K-Akt-mTOR signaling pathway [45] whose overactivity may contribute to resistance of ER-positive cancers to endocrine therapy. It has been also shown that sulforaphane at 10 μM concentration potentiated anti-proliferative activity of tamoxifen used at 1 μM concentration in the ER-negative MDA-MB-231 cells, which was further enhanced by green tea polyphenols due to chromatin modification and reactivation of the ER expression [38]. The purpose of our study was to experimentally test the hypothesis that ITC at relatively low concentration may intensify the anti-proliferative effect of 4-hydroxytamoxifen on ER-positive breast cancer cell lines. We used two structurally related ITC and breast cancer cell lines which differ in the amount of ER and HER2 receptors as well as generation times.

We demonstrate that combinations of 4-hydroxytamoxifen at a low inhibitory concentration and any isothiocyanate at concentrations lower than their IC_50_ values inhibit cell proliferation of ER-positive human breast cancer cells more efficiently than any compound used alone. The results of the MTT tests show that BT-474 cell line was the least sensitive to the drug or ITC. It could be related to a slower growth rate of BT-474 cells. Their doubling time exceeds 100 h, while doubling rate for MCF-7 cells is about 29 and T47D—about 32 h. Moreover, these cell lines differ in the expression levels of growth factor receptor genes. BT-474 cell line is characterized by comparable level of ER (11.3 fmol/mg protein) but much higher level of HER2/neu (12,256 fmol/mg protein) when compared to MCF-7 cells (ER—11.5 fmol/mg protein and HER2—297 fmol/mg protein) or T47D (ER—16.7 fmol/mg protein and HER2—165 fmol/mg protein) [34]. Preclinical research and clinical trials have revealed that magnitude of the response to treatment with tamoxifen correlated with the ER level and its crosstalk with other growth factor receptors, such as HER2 [12, 54] which may explain higher resistance of BT-474 cells to treatment with 4-hydroxytamoxifen or ITC. However, despite this, viability of BT-474 cells was significantly decreased in experimental variants treated with a combination of phytochemicals (sulforaphane or erucin) and 4-hydroxytamoxifen compared to cells treated with any compound alone. Moreover, combinations of 4-hydroxytamoxifen at a low concentration with SFN or ERN efficiently inhibited clonogenic potential of the breast cancer cells. It is worth noting that ability of cells to proliferate was significantly reduced after only 8 days of treatment with combinations and subsequent cessation of therapy. Longer therapy might have a more pronounced effect.

Several studies have shown that high concentrations of erucin and sulforaphane induce apoptosis-associated proteolytic cleavage of poly(ADP-ribose) polymerase in different cancer cell lines [39, 44, 56]. Here we demonstrate that combinations of sulforaphane or erucin with 4-hydroxytamoxifen at low concentrations induce apoptosis of breast cancer cell lines more efficiently than any compound used alone. Substantial drop in Bcl-2/Bax ratio upon combined treatment, as well as an increased level of ADRP, the marker of mitochondrial stress-mediated lipid droplet formation, points toward involvement of mitochondrial pathway in apoptosis induction in each cell line; however, death receptor pathway cannot be excluded.

We also determined the effect of single as well as combined treatment with ITC and 4-hydroxytamoksifen on survivin level in all three cell lines. Survivin inhibits apoptosis and promotes cell proliferation and angiogenesis. This protein is undetectable in majority of normal adult tissues; however, it is often overexpressed in tumors, including breast cancers (in 70–90 % of cases) [3, 33]. High level of survivin correlates with progression of cancer and resistance to therapies [2, 63]; thus, its targeting might improve efficacy of chemo- or radiotherapies [33]. We observed that SFN, ERN or 4-hydroxytamoxifen downregulates survivin to a similar extent in T47D and MCF-7 cells and combinations of the drug and ITC potentiate this effect. Interestingly, in BT-474 cells, ITC alone elevated the survivin level when compared with controls, which might explain lower sensitivity of this cell line to SFN or ERN. However, their combination with the 4-hydroxytamoxifen decreased the survivin level. Here, the net result of combined treatment on cell viability might be caused not only through survivin but also by Bcl-2 level modulation. The Bcl-2 level changed in an opposite manner to that of survivin: It was elevated by 4-hydroxytamoxifen and reduced by ITC and their combinations with the drug. Levels of anti-apoptotic proteins are regulated by multiple pathways; thus, the impact of chemicals used by us on Bcl-2 or survivin expression might be related to the genetic background of the cells used.

We also observed that ITC and 4-hydroxytamoxifen induce processing of the LC3 protein, a specific marker of autophagosomes [29], especially when used in combinations. Autophagy is one of the most important strategies for protein degradation and protein/organelle quality control mechanism, and during cellular stress, it provides nutrients to support metabolism [14]. Accumulated evidence suggests that a basal or slightly increased level of autophagy can protect cells against apoptosis; however, prolonged or intensive autophagy can lead to cell death [7, 46]. For instance, SFN-induced autophagy in prostate as well as breast cancer cells played a protective role [22, 31] but benzyl isothiocyanate-induced autophagy in breast cancer cells contributed to their death [62]. The role of autophagy induced by tamoxifen or its metabolites in breast cancer cells is controversial. Initial studies documented that tamoxifen and 4-hydroxytamoxifen caused an autophagic death (also referred to as type II cell death) of MCF-7 cells [9, 10]. However, more recent data indicate that in response to 4-hydroxytamoxifen, only a small part of cell population dies, while majority of cells are arrested in growth and viable. Interestingly, elevation of autophagy in living cells decreased drug-induced death, while autophagy inhibition resulted in a more robust caspase-dependent death of MCF-7 cells [52]. Moreover, the authors have shown that in tamoxifen-resistant MCF-7 cells, autophagy inhibition sensitized these cells to the antiestrogen [52]. In our study, the combined treatment with ITC and 4-hydroxytamoxifen also induced protective autophagy in MCF-7 and T47D cell lines, while in BT-474 cells contributed to their lower survival. The reason for the observed differences is not known at this moment; however, it was previously reported that BT-474 cells contain a high basal level of autophagosomes [16], which is in agreement with our observation showing an increased LC3-II level even in nontreated cells. Thus, it is possible that upon treatment with ITC and 4-hydroxytamoxifen autophagy reaches the threshold necessary for cell death.

Finally, we show that SFN or ERN efficiently sensitized tamoxifen-resistant variants of MCF-7 and T47D cells to 4-hydroxytamoxifen. It seems that synergistic activity of ITC and the drug used at a low concentration (0.5 μM) is ER dependent, which is evidenced by the fact that our T47D tamR and MCF-7 tamR cells retained this receptor and are sensitive to the combined treatment, while in the MDA-MB-231 cells, which are devoid of ER, ITC do not abrogate resistance to 0.5 μM 4-hydroxytamoxifen. On the other hand, ITC, especially erucin, might potentiate the cytotoxic activity of the drug used at 5 μM concentration. However, high concentrations of tamoxifen have been shown to have an ER-independent nongenomic effect in ER-negative breast and other cancer cells [18, 30, 64]. Thus, it is possible that the mechanism of sensitization to 4-hydroxytamoxifen by ICT might be related to the drug dose.

Important question arises when coming to in vivo use of combined therapy: whether micromolar concentrations of ITC are achievable in humans. In case of SFN, it has been shown that in human subjects who ingested 100 g of broccoli as a soup, its peak plasma concentrations reached 2.2 μM [20]. A more recent study shows that 3 h after consumption of broccoli sprouts providing 200 μmol SFN, plasma levels of total SFN metabolites reached about 2 μM concentration [4]. In rats, after an oral dose of 50 μmol of SFN was delivered, its plasma concentration peaked around 20 μM at 4 h after dosing [23]. The bioavailability of erucin is not known at this time. However, SFN and ERN are structurally similar and reveal similar pharmacokinetics. Moreover, interconversion of SFN to ERN has been reported in rats and humans [32, 61]. Concentration of these ITC in plasma or tissue after multiple dosing of pure compounds can be higher than after one dose of plant extract; however, it has not been investigated so far. In case of the drug, it has been shown that clinically relevant steady-state plasma concentrations of tamoxifen and its biologically active metabolites can be as high as 5 μM in patient sera [47].

In conclusion, our data indicate that sulforaphane or erucin, used at relatively low concentrations, potentiate anticancer activity of 4-hydroxytamoxifen. This effect is mediated by downregulation of anti-apoptotic proteins such as Bcl-2 and survivin and in consequence by induction of cell death. This strategy allows for using tamoxifen at lower doses, hence decreasing the level of its toxicity and improving the risk–benefit profile of this agent. Moreover, it might protect against acquisition by cancer cells the drug-resistant phenotypes during therapy.
